# Qualitative overview of indoor radon surveys in Europe

**DOI:** 10.1016/j.jenvrad.2019.04.010

**Published:** 2019-08

**Authors:** Gordana Pantelić, Igor Čeliković, Miloš Živanović, Ivana Vukanac, Jelena Krneta Nikolić, Giorgia Cinelli, Valeria Gruber

**Affiliations:** a "Vinča" Insitute of Nuclear Sciences, University of Belgrade, Serbia; b European Commission, Joint Research Centre (JRC), Ispra, Italy; c Austrian Agency for Health and Food Safety, Department of Radon and Radioecology, Linz, Austria

## Abstract

The revised European Directive from 2013 regarding basic safety standard oblige EU Member States to establish a national action plan regarding the exposure to radon. At the same time, International Atomic Energy Agency started technical projects in order to assist countries to establish and implement national radon action. As a consequence, in recent years, in numerous countries national radon surveys were conducted and action plans established, which were not performed before. In this paper, a qualitative overview of radon surveys performed in Europe is given with a special attention to the qualitative and conceptual description of surveys, representativeness and QA/QC (quality assurance/quality control).

## Highlights

• Literature overview of indoor radon surveys performed in Europe.
• Analysis of survey design and representativeness of indoor radon surveys.
• Analysis of measurement techniques used in indoor radon surveys.
• Analysis of sampling procedure, sampling number and type of locations in indoor radon surveys.
• Analysis of quality assurance and quality control in indoor radon surveys.

## 1 Introduction

Natural radioactivity is the main source of population exposure to ionising radiation. More than 80% of exposure comes from the natural radioactivity. Radon and its progenies contribute with more than 50% to annual effective dose received from all sources of ionising radiation (UNSCEAR, 2008).

Radon is a radioactive noble gas, with no stable isotopes. Three naturally occurring isotopes ^222^Rn, ^220^Rn and ^219^Rn, are products of the decay of radium that originates from the decay chain of three primordial decay series ^238^U, ^232^Th and ^235^U, respectively. The relative importance of radon isotopes increases with an increase of their half-lives and their relative abundance. Due to the short half-life of ^219^Rn (T_1/2_ = 3.98 s) compared to ^222^Rn (T_1/2_ = 3.82 d), and isotopic ratio of ^235^U/^238^U = 0.0072, ^219^Rn is always ignored. Although ^220^Rn (in text referred as thoron) is relatively short-lived (T_1/2_ = 55.8 s) compared to ^222^Rn (in text referred as radon) and hence can travel much smaller distances, there are regions with exceptionally high ^232^Th/^238^U ratios leading to a much higher thoron concentration that cannot be neglected.

Being chemically inert, with a lifetime that is long compared to a breath rate, most of the inhaled radon is exhaled rather than decaying in human respiratory system. On the other hand, short-lived radon progenies are solids and tend to attach to surfaces, mainly aerosols. When inhaled they stick to epithelial surfaces and due to a short lifetime their decay sequence finishes before lungs can clean them out, irradiating therefore sensitive surfaces of bronchi and lungs. Hence, health hazards related to radon issue are not caused directly by radon, but by its short-lived progenies.

Historically speaking, radon problem dates from XV century when high death rate due to lung diseases has been observed among silver miners in the regions of Scneeberg in Saxony and Jachimov in Bohemiaas (Paracelsius, 1567). The illness was identified as lung cancer 4 centuries later by Haerting and Hesse (1879). A year after the Dorn's discovery of radon, Elster and Geiter have measured high radon concentration in air in mines of Schneeberg and Jachimov (Elster and Geitel, 1901), but high radon concentration was still not connected with lung cancer. Finally, Rajewsky and collaborators have assumed a link between high radon concentration and lung cancer in 1940 (Rajewsky, 1940) and afterwards in 1951, Bale suggested that radon short-lived progenies could be the main cause of lung cancer (Bale, 1951). From the analysis of the first cohort studies conducted between uranium miners in America (Lundin et al., 1971) and Czechoslovakia (Sevc et al., 1976) it was concluded that there is a monotonic increase of a lung cancer risk with the cumulative exposure to radon progenies. Numerous miner studies were followed, mainly based on above-mentioned studies, and in 1988 International Agency for Research on Cancer has ascribed radon as a human carcinogen (IARC, 1988).

The results of the first indoor radon survey, conducted in Sweden, were published in 1956, and among 225 investigated houses, a few of them had very high radon concentration (Hultqvist, 1956). In that time, the international scientific community considered these findings as a local Swedish problem. Only after 20 years, indoor radon concentration was investigated more seriously in a number of countries and national radon programmes and regulations had been introduced (UNSCEAR, 2000).

Based on those investigations, recent radon pooling studies performed in China, Europe and North America have unambiguously shown connection between indoor radon concentration and lung cancer (Darby et al., 2006; Krewski et al., 2006; Lubin et al., 2004). Based on these studies, radon was identified as the second leading cause of lung cancer after cigarettes, being responsible for 3%–14% of all lung cancers (WHO, 2009).

The Joint Research Centre (JRC) of the European Commission decided to embark on a European Atlas of Natural Radiation (EANR) (De Cort et al., 2011), in line with its mission, based on the Euratom Treaty (European Union, 2016), which is to collect, validate and report information on radioactivity levels in the environment. The Atlas is a collection of maps of Europe displaying the levels of natural radioactivity caused by different sources: from cosmic radiation to terrestrial radionuclides. The digital version of the EANR is available on line at https://remon.jrc.ec.europa.eu/(Cinelli et al., 2019) and the publication is foreseen in 2019. As a first task, the JRC started to prepare a European Indoor Radon Map (EIRM), given its great radiological importance (WHO, 2009). A first overview of indoor radon surveys in Europe has been performed in 2005 by Dubois (2005). The review of surveys has shown heterogeneity of data, starting from the survey strategies, sampling strategy, measurement techniques, measurement duration and season. Therefore, a huge effort has been taken to summarise data of indoor radon concentrations from different countries and to integrate them in a homogeneous way to produce a European map of indoor radon levels using a 10 km × 10 km grid cells (Dubois et al., 2010).

The exposure of members of the public and of workers to indoor radon is now explicitly taken up in the scope of Basic Safety Standards (BSS) Directive – Directive 2013/59/Euratom laying down basic safety standards for protection against the dangers arising from exposure to ionising radiation (Article 2 (2d)) (European Union, 2013). According to the 2013 BSS directive all member states are required to have a radon action plan and inform the population about their radon levels. Radon activities and radon surveys therefore were started or repeated in several countries in the last years and are still ongoing and maybe will be also increased in the next years. For non-EU-member states also IAEA BSS require radon surveys and IAEA guidelines how to perform radon surveys exist (IAEA, 2011).

Recently, a JRC report based on literature review of indoor radon surveys in Europe was given within the framework of MetroRADON project (Pantelić et al., 2018). Based on data from the report, this overview was prepared aiming to give an updated qualitative overview of radon surveys performed in European countries using literature data, with focus on the data which were not included in other survey overviews. Therefore, special attention is given to the qualitative and conceptual description of surveys such as types of surveys and their representativeness, sampling strategies and measurement techniques, applied corrections, interpretation of survey results and dealing with thoron issue.

The literature overview has shown that many sources do not present sufficient data on survey design and survey results, so in many cases the number of identified answers is lower than the number of surveys that was studied in this research.

## 2 Survey design and representativeness

Although the main source of indoor radon is soil subjacent to the dwelling, knowing only soil characteristics is not enough to obtain a reliable prediction of indoor radon concentration of specific dwelling, due to numerous factors influencing radon concentration. Since it is not feasible to perform a measurement for each dwelling it is important to carefully design radon survey in order to obtain representative distribution of radon concentration in dwellings. (IAEA, 2013).

Performing a truly representative indoor radon survey is rather difficult. In order to achieve truly representative survey, it is necessary to have a complete list of dwellings, which is seldom available, from which random selection of dwelling should be chosen. Any deviation from pure random sampling can cause biases (IAEA, 2013). It was shown that volunteer measurements could be biased due to the over-sampling in radon priority areas (Burke and Murphy, 2011).

This type of survey based on random sampling is population-weighted survey, since more dwellings will be sampled in densely populated region. Another type of survey is geographically based radon survey in which a territory is divided into geographical units, such as rectangular grids of certain area or administrative boundaries (strata). Sampling within each geological unit should be representative for the population distribution within that unit. Therefore, with carefully designed survey, representativeness of both approaches can be achieved (IAEA, 2013).

The overview of radon surveys presented in this paper was conducted in such a way to identify the survey covering the largest territory for each European country – preferably a national survey. If a national survey was identified, no regional surveys were considered. If more than one national survey was found, then the most recent one was considered, or the most recent publication that covered results from previous surveys as well. In some cases, more than one regional survey was considered if they did not overlap significantly. Some special surveys were considered to point out different methodologies. It is likely that more recent surveys exist in some countries, but no literature was available. Some surveys continued past the publication date of the paper or document that was analysed for the purposes of this research, but the analysis is limited only to the published results.

Indoor radon surveys have been conducted in most European countries – existing surveys were identified, through extensive literature research, for all countries except Andorra, Liechtenstein, Monaco, San Marino and Vatican. At least one survey was conducted in each European Union (EU) member country. In some cases, scientific papers and other sources reporting radon concentrations aggregated results of several different surveys. For the purposes of this paper, if the overall coverage is national, it will be considered that a national survey was conducted, for brevity purposes.

National surveys were conducted in 22 EU countries: Austria (Friedmann, 2005) Croatia (Radolić et al., 2006), Czech Republic (Hůlka, 2014; Slezáková et al., 2013; Thomas et al., 2004), Denmark (Andersen et al., 2007, 2001), Estonia (Pahapill et al., 2003), Finland (Valmari et al., 2010; Weltner et al., 2002), France (Gambard et al., 2000; Rannou et al., 2006), Greece (Nikolopoulos et al., 2002), Hungary (Hámori et al., 2006; Nikl, 1996), Ireland (Dowdall et al., 2017; Fennell et al., 2002), Italy (Bochicchio et al., 2005; Carelli et al., 2009), Lithuania (Morkunas and Akelbrom, 1999), Luxembourg (Kies et al., 1997), Malta (Baluci et al., 2013), Netherlands (Lembrechts et al., 2001; Stoop et al., 1998), Poland (Przylibski et al., 2011), Portugal (Faisca et al., 1992), Slovakia (Vicanova et al., 1998; Vladár et al., 1996), Slovenia (Humar et al., 1995; Križman et al., 1996), Spain (Sainz Fernández et al., 2017), Sweden (Swedjemark, 2002; Swedjemark et al., 1993), United Kingdom (Daraktchieva et al., 2015; Miles et al., 2007, 2011). Only regional surveys were identified in 5 member states: Belgium (Cinelli et al., 2011; Poffijn et al., 1994; Tondeur et al., 1997; Zhu et al., 2001, 1998), Bulgaria (Ivanova et al., 2013), Cyprus (Anastasiou et al., 2003; Theodoulou et al., 2012), Germany (Kemski et al., 2004, 1996), Latvia (Dambis, 1996), Romania (Cucoş (Dinu) et al., 2017). Outside the EU, national surveys were conducted in Azerbaijan (Hoffmann et al., 2017), Belarus (Yaroshevich et al., 2012), Iceland (Jónsson et al., 2015), Macedonia (Stojanovska et al., 2012), Montenegro (Vukotic et al., 2018), Russia (Yarmoshenko et al., 2015), Serbia (Udovičić et al., 2016), Switzerland (Kropat et al., 2014), Ukraine (Pavlenko et al., 2014) and Norway (Jensen et al., 2004). Only regional surveys were identified for Albania (Bode Tushe et al., 2016), Armenia (IAEA, 2014), Bosnia and Herzegovina (Ćurguz et al., 2015; IAEA, 2014), Georgia (IAEA (International Atomic Energy Agency), 2014), Kazakhstan (Fyodorov et al., 2014), Moldova (Ursulean et al., 2013), and Turkey (Can et al., 2012; Köksal et al., 2004).

The number of measurement locations in the surveys covered in this paper differs by 4 orders of magnitude. This data are not always reliably identifiable from the references. In some cases, more than one measurement was performed per location, sometimes at the same time in different part of the building, sometimes at different time. However, in order to compare the surveys, only unique locations with valid measurement results were counted, as reported by the survey authors. Some of the surveys continued after the last publication of the results, so the numbers of measurement locations could be higher.

The minimum number of locations was selected in Malta national survey – 85 (Baluci et al., 2013) At the other end of the spectrum, radon measurements from more than 500,000 locations are available in UK (Daraktchieva et al., 2015; Miles et al., 2007, 2011). There are at least 5 countries besides UK with more than 50,000 measurement locations – Russia (Yarmoshenko et al., 2015), Czech Republic (Dubois et al., 2010), Switzerland (Kropat et al., 2014), Finland (Valmari et al., 2010) and Norway (Jensen et al., 2004).

**Fig. 1:**
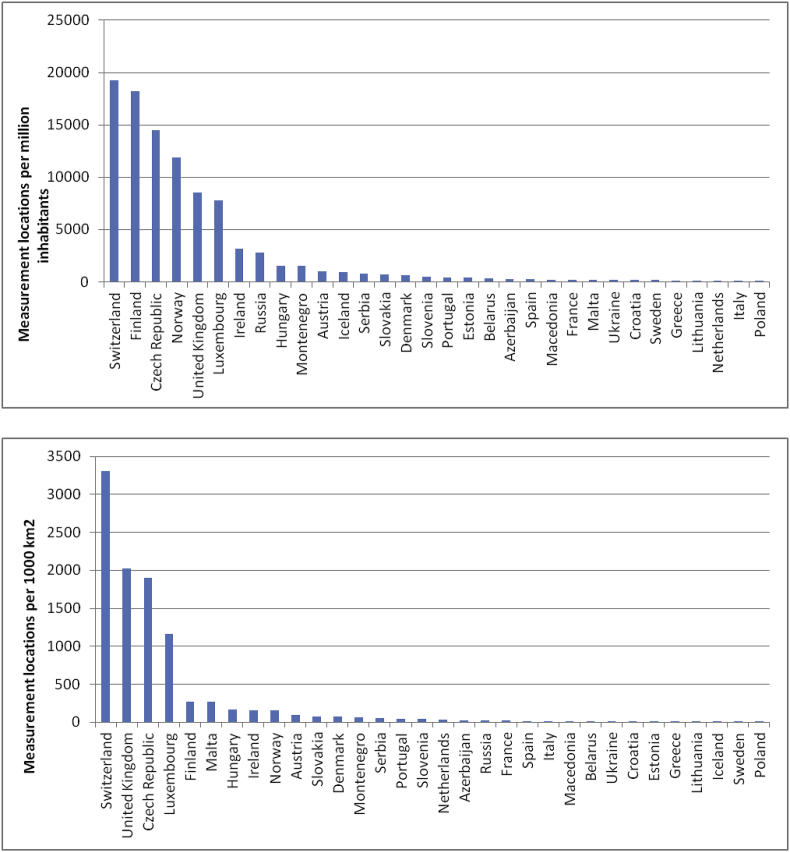
Number of measurement locations per million inhabitants (top figure) and per 1000 km^2^ (bottom figure).

**Fig. 2:**
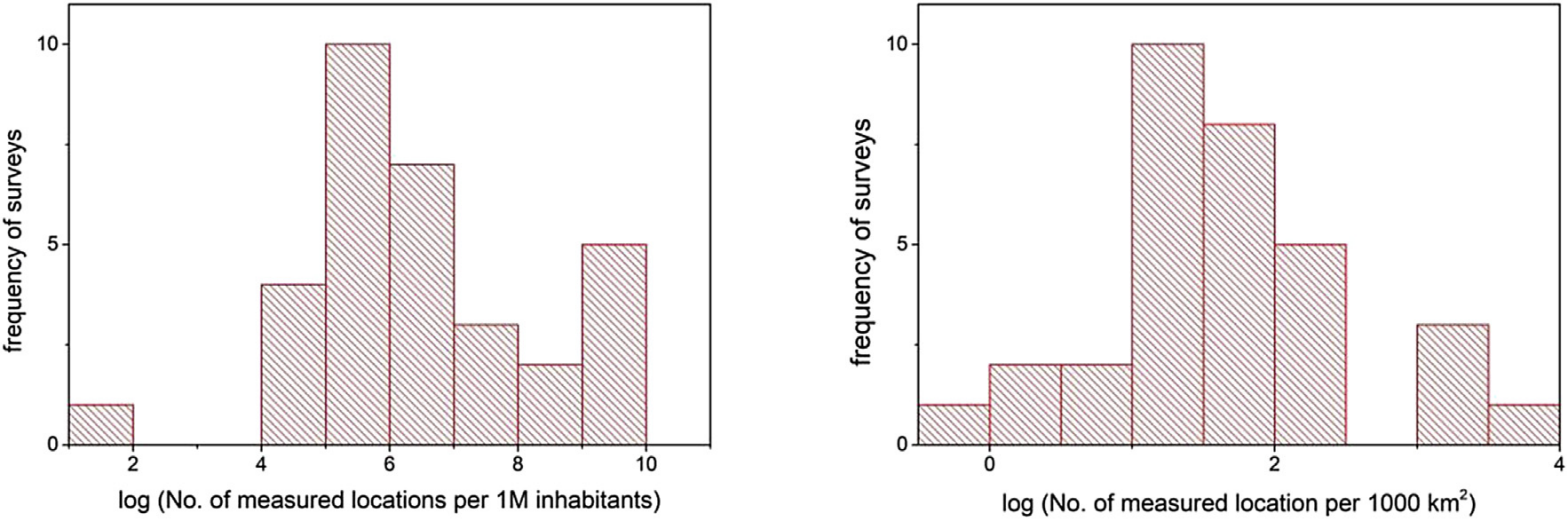
Frequency distribution of the number of measured locations normalised: per 1 million inhabitants (left figure) and per 1000 km^2^. X-axis is given is natural logarithm scale.

Dividing the number of measurement locations by country territory or population can provide another perspective. The results are graphically shown in Fig. 1. For this graphics, only national surveys are represented. Population data are taken from Google public data for the year that is at the middle of the survey period, or the nearest year for which there are available data. Country area is in most cases excluding overseas territories (e.g. Greenland and Svalbard) but in other cases, territory outside of Europe is taken into account since the survey covers that territory (e.g. Russia and Azerbaijan). The ratios should be considered only as approximations. Frequency distribution of the natural logarithm of the number of measured locations normalised: per 1 million inhabitants and per 1000 km^2^ are presented on the left hand side, and on the right hand side of Fig. 2.

In both cases, Switzerland, Finland, UK and Czech Republic are in top 5, as is the case when the absolute number of measurement locations is used. However, Russia is in the bottom half if the area is considered, and Malta is comparable with Finland.

In almost all indoor radon surveys, the great majority of measurement locations were dwellings. However, other measurement locations were also selected in some surveys: schools and kindergartens (Iceland (Jónsson et al., 2015), Luxembourg (Kies et al., 1997), Russia (Zhukovsky et al., 2012), Slovakia (Vicanova et al., 1998; Vladár et al., 1996), Slovenia (Humar et al., 1995; Vaupotic et al., 1992), Ukraine (Pavlenko et al., 2014)), industrial buildings and workplaces (Azerbaijan (Hoffmann et al., 2017), Moldova (Ursulean et al., 2013), Luxembourg (Kies et al., 1997), Italy (Carelli et al., 2009)), swimming pools (Iceland (Jónsson et al., 2015)), spa buildings and caves (Slovakia (Vicanova et al., 1998)) and underground Telecom inspection rooms (Italy (Carelli et al., 2009)).

Regarding the most recent surveys, many countries have published survey results in the previous 10 years, including Albania (Bode Tushe et al., 2016), Azerbaijan (Hoffmann et al., 2017), Belarus (Yaroshevich et al., 2012), Bulgaria (Ivanova et al., 2013), Iceland (Jónsson et al., 2015), Kazakhstan (Fyodorov et al., 2014), Malta (Baluci et al., 2013), Romania (Cucoş (Dinu) et al., 2017), Serbia (Udovičić et al., 2016), Turkey (Köksal et al., 2004), Ukraine (Pavlenko et al., 2014). Most of them were conducted, under the technical cooperation programmes with IAEA, aiming to develop policies and strategies according to requirements of Basic Safety Standards (IAEA, 2011).

On the other hand, countries with long history in radon surveys often do not have any recent results published in the available literature. It is, however, probable that the indoor radon measurements are still on-going in these countries. Examples of such countries are United Kingdom, Austria, Czech Republic, Norway, Sweden, France, Hungary.

Survey goals were in most cases to produce an indoor radon map (i.e. to determine a geographical distribution of indoor radon levels), to identify radon priority areas, to assess the effective dose, to determine national mean concentration and to provide inputs for national legislation or action plans. In several cases, no map was created, but the descriptive statistics was performed for territorial units within the country. Regional studies were often conducted in the previously identified radon priority areas. In the study conducted by Carelli et al. (2009), the goal was to test a novel mapping method, and in the study conducted by Slezáková et al. (2013), to evaluate long term variability of radon concentrations.

The European Indoor Radon Map is based on the average indoor radon concentrations within 10 km × 10 km grid cells (Dubois et al., 2010). This sampling strategy is more prevalent in newer studies and it can be expected that it will be more so in the future for radon mapping purposes, which is a requirement of the 2013BSS (European Union, 2013). However, there is a large diversity within sampling strategies in existing radon surveys. In many countries, territory was subdivided into administrative units (Denmark (Andersen et al., 2007, 2001), France (Rannou, 1990) and Netherlands (Lembrechts et al., 2001; Stoop et al., 1998)) or grid cells – 10 km × 10 km (Albania (Bode Tushe et al., 2016), Azerbaijan (Hoffmann et al., 2017), Hungary (Nikl, 1996), Ireland (Fennell et al., 2002), Romania (Cucoş (Dinu) et al., 2017) and Spain (Sainz Fernández et al., 2017)), 5 km × 5 km (Malta (Baluci et al., 2013)), 1 km × 1 km (Cyprus (Theodoulou et al., 2012) and United Kingdom (Daraktchieva et al., 2015; Miles et al., 2007, 2011)) or even 0.5 km × 0.5 km (Montenegro (Vukotic et al., 2018)). In case of Poland, country was divided into geological regions (Przylibski et al., 2011). In other cases, density of measurement points was correlated to the population density or was higher in previously identified radon priority areas. Finally, in the study conducted by Istituto Superiore di Sanità building network of Telecom Italia was used (Carelli et al., 2009).

### 2.1 Representativeness

In most cases, authors of reviewed surveys did not go in details about survey design and its representativeness. Therefore authors of this overview of surveys did not try to estimate whether some surveys were representative or not. Instead, an overview to what extent representativeness was discussed in reviewed papers is given.

In most surveys, random sampling within each grid cell, territorial unit or the whole country was used. However, many surveys were based on volunteers within special cohorts (physics teachers, students, civil servants on municipal level etc.) or measurements in government buildings, usually schools or kindergartens. Some surveys based on volunteers could be biased toward higher concentrations since people suspecting to live in higher indoor radon concentration tend to volunteer more. Also, volunteers, such as students, could represent a specific part of population that is not necessarily representative of the whole population.

In Iceland it was underlined that although broad distribution of sample points was achieved, sampling locations were not random (Jónsson and Theódorsson, 2003; Jónsson et al., 2016).

In Estonian survey, it was underlined that a representative number of dwellings was used and that obtained results are representative for detached houses and flats on the ground floor for multiapartment buildings (Pahapill et al., 2003).

In Germany, a standardised procedure for radon and permeability measurements was developed to assure regional representativeness. Number of measurement per sampling area depended on the variability of geological patterns in the area (Kemski et al., 1996).

In population-weighted survey performed in Macedonia, representativeness was obtained by random selection of houses, covering all regions (Stojanovska et al., 2012).

Data obtained from the questionnaires sent to inhabitants during the first Hungarian radon survey were compared with data from Central Statistical Office in order to check the representativeness of the sample (Nikl, 1996). The second survey in Hungary was based on volunteers where teachers facilitated distribution of the detectors. It was concluded that due to large measurements performed, sampling could be considered representative (Hámori et al., 2006).

Due attention on representativeness of both national radon surveys in Ireland was given. By designing the first survey it was concluded that at least 5 dwellings per 10 km^2^ grid square should be selected. In order to ensure at least this sample size, 70 householders per grid square were randomly selected from the Register of electors (Fennell et al., 2002). The second survey was carefully designed to assure radon measurements in the sample of homes are representative of radon risk and geographical location. By random selection from Geodirectory – a database of Irish postal addresses identified by geographical coordinates, a representative sample of dwelling types is provided. Finally, the representativeness of the grid squares was checked by the goodness of fit between distributions of geographic regions and risk categories (Dowdall et al., 2017).

The Italian national indoor radon survey was designed to obtain a representative estimate of the radon distribution in dwellings. Representative number of dwelling was selected in two stages: the first stage was a simple random sampling of towns over 100000 inhabitants and clustered and then random sampling of smaller towns. In the second step, dwellings were randomly sampled within each town with the sampling proportion of 1/4000 (Bochicchio et al., 2005). In the most recent Italian survey, conducted in the workplaces and employees’ home of national telecom company, that encompassed about 7000 dwellings, representativeness was checked in details by comparing characteristics of dwellings with data from the latest National Census (Antignani et al., 2013).

It is estimated by Daraktchieva and coauthors that surveys performed in UK are seldom representative since many measurements targeted the areas where high radon concentrations were expected. The first UK survey performed by Wrixon and collaborators was the only population weighted survey (Daraktchieva et al., 2015; Wrixon et al., 1988).

In the report of Swedish Residential Radon Project, it is mentioned that a representative sample of Swedish housing stock was performed during 1976 and 1988 (Swedjemark, 2002).

In Austrian survey, dwellings were selected randomly from the telephone register to avoid a biased sample. In case of refusal, another house was randomly selected. Measurements were populated weighted, with 1 in 200 homes selected for the sample (Friedmann, 2005).

Ivanova et al. have emphasised that the main goal in the regional Bulgarian radon survey was to choose representative districts in order to obtain representative results of the indoor radon. Number of dwelling for each district was population weighted, but considering also a spatial distribution (Ivanova et al., 2013).

In Czech Republic, there is a continuous radon program going from early eighties with more than 150000 measurements. Representativeness is not directly discussed. It was mentioned only that first indoor radon survey performed in 1992/93 was representative (Hůlka and Thomas, 2004).

Radon survey in Greece was administratively designed. Sampling density was 1 per 1000 dwellings. A door-to-door approach was applied in order to minimise nonresponse and bias (Nikolopoulos et al., 2002).

Representativeness of radon survey in Lithuania was not discussed directly. Nevertheless, it is mentioned that random sampling of detached house was applied with density of one house in 1096 in rural areas and one house in 1120 in urban areas (Morkunas and Akelbrom, 1999).

Representative national survey of Croatia was obtained by random sampling of thousand addresses (Radolić et al., 2006). In Montenegro, an advice from construction expert was obtained in order to identify houses that could be considered as representative. One such house has been then identified in each grid square and selected for radon measurements (Vukotic et al., 2018).

Based on one of the regional surveys conducted in Serbia, a question was raised whether indoor radon survey in Serbian schools could produce results representative for radon exposure of the general population (Žunić et al., 2010a, Žunić et al., 2010b). Based on these results, in regional survey of indoor radon, thoron and its progenies in schools in Bosnia and Herzegovina, it was stated that representative measurements were performed due to correlation of primary schools with the number of residents (Ćurguz et al., 2015).

In some surveys (Belgium, Finland and Switzerland), that have over-sampled areas, different techniques, such as declustering, were applied to achieve regional representativeness (Kropat et al., 2014; Valmari et al., 2010; Zhu et al., 1998).

In Cyprus survey, no direct discussion about representativeness is present. Nevertheless, it was mentioned that house owners were approached by phones to get their agreement. Although measurement per dwelling lasted only for 2 days, it is mentioned that, due to constant weather conditions, there is no reason for seasonal corrections. Finally, authors have mentioned representative overview of results, by their classification in different regions (Anastasiou et al., 2003).

In national radon survey of Iceland, volunteers were sought via webpage or by phone and therefore sampling locations were not randomly selected. Nevertheless, they tried to select dwellings following population density distribution (Jónsson et al., 2015).

From 2013 a comprehensive radon survey is on-going in Romania (Cucoş (Dinu) et al., 2017). Although, representativeness was not mentioned in the analysed paper, it is underlined that survey protocol designed on the basis on the European Indoor Radon Map (Tollefsen et al., 2014). At each 10 km × 10 km grid cell, deferent number of detectors, from 3 to 15 has been deployed depending on population density (Cucoş (Dinu) et al., 2017).

The Spanish indoor radon map was constructed based on a few surveys. Grid was generated according to the European Indoor Radon Map. The last survey was designed in such a way to add missing measurements in different grid cells in order to fulfil several criteria: surface criterion, population criterion, MARNA criterion increased number of measurement in areas with high radon potential, and lithostratigraphic criterion. Measurement locations at each cell were selected randomly. (Sainz Fernández et al., 2017).

For performing a representative survey, it is not sufficient only to have random, unbiased sampling of dwellings, but also appropriate measurement techniques should be used, appropriate measuring location. If the goal is to have a representative survey, it should also be part of the survey to test at the end, to what extend representativeness was reached (e.g. by comparison to national census data) that this in most or the surveys is not done yet (Antignani et al., 2013).

## 3 Measurement techniques

There are numerous techniques for radon measurement, which can be performed by direct measurement of radon, so called "radon alone" measurement or indirectly by measurement of radon progenies with or without radon itself. Since radon and some of its progenies - ^218^Po, ^214^Po and ^210^Po - are alpha emitters, while ^214^Pb, ^210^Pb, ^214^Bi and ^210^Bi are beta emitters, and their decay is mostly followed by gamma-ray emission, radon measurements can be performed by detection of either alpha, beta or gamma rays. Some widely used techniques are: solid state nuclear track detectors, ionisation chambers and proportional counters, scintillators, semiconductors with surface barrier, gamma spectrometry, and adsorption.

A strong variation of radon concentrations in time was found. Roughly speaking, one can identify 2 types of variations of indoor radon concentrations: diurnal and seasonal. On daily basis, radon concentrations are higher during the night and early morning, while they decrease during the day. Radon concentrations are in general higher during the heating season, compared to non-heating season. Therefore, measurements should be long enough to enable averaging these variations.

Depending on the duration, measurements can be: 1) instantaneous measurements in which sample of radon gas is collected in the time interval of the order of minutes (known as grab sampling); 2) continuous measurements in which a radon concentration is continuously monitored with the radon concentration integrated over a certain period of time (of the order of minutes or hours); and 3) integrated measurements in which radon is measured and therefore averaged over a long period of time (of the order of days or months).

Thus, the choice of measurement technique depends on the purpose of radon measurement and since for radon surveys the goal is to obtain an average annual radon concentration the most appropriate would be long term measurement. (IAEA, 2013).

Indoor radon surveys in investigated European countries were performed with passive measurement techniques except in one country (Cyprus). Only in Cyprus, the indoor measurements were carried out by using a high sensitivity active portable radon monitors - RADIM3A (Anastasiou et al., 2003; Theodoulou et al., 2012).

**Fig. 3:**
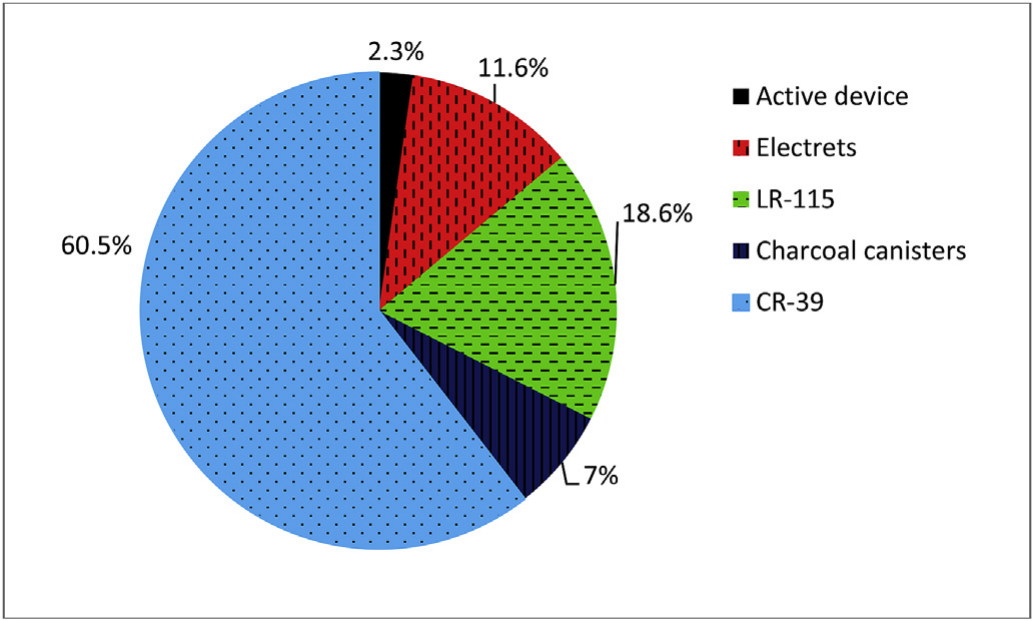
Overview of used techniques for radon surveys.

An overview of used techniques for radon surveys is shown in Fig. 3. From 42 countries which were covered by this survey, passive ***electrets*** detectors were used in indoor radon surveys in five countries: Austria (Friedmann, 2005), Hungary (Nikl, 1996), Latvia (Dambis, 1996), Lithuania (Morkunas and Akelbrom, 1999), and Switzerland (Kropat et al., 2014). Different kind of passive track detector systems based on solid state track detectors ***LR-115*** were used in eight countries: Belarus (Yaroshevich et al., 2012), Croatia (Radolić et al., 2006), Czech Republic (Slezáková et al., 2013), France (Gambard et al., 2000; Rannou, 1990; Rannou et al., 2006), Italy (Bochicchio et al., 2005), Malta (Baluci et al., 2013), Portugal (Faisca et al., 1992), and Ukraine (Pavlenko et al., 2014, 1997). In two covered countries, indoor radon concentrations were measured by gamma ray spectrometry (NaI(Tl) or HPGe detectors) of exposed ***charcoal canisters*** in Austria (Friedmann, 2005), Belgium (Cinelli et al., 2011; Tondeur et al., 1997; Zhu et al., 2001).

Literature survey showed that the most commonly used measuring technique (in more than 60%) is alpha track detectors ***CR-39*** (polyallyl diglycol carbonate), etched with NaOH after exposure and track counting by different approaches.

Several countries used other kind of track detectors without specification what films were used, like Azerbaijan – Gammadata-Landauer type (Hoffmann et al., 2017), Finland – Alpha track detectors (Valmari et al., 2010; Weltner et al., 2002), Germany – solid state nuclear track detector (Kemski et al., 2004).

Results of a literature survey, regarding indoor radon measurement campaigns, also showed that in some countries different measurement techniques were combined, either in one survey or during the different conducted surveys.

### 3.1 Single measurement design and evaluation

The measurement time is mainly conditioned by the selected measuring technique. For indoor radon measurements by highly sensitive active portable monitors (in Cyprus) instrument was adjusted to record the data every 2 h over the 24 h period (or 2–4 h over 48 h). Drought-free areas in the sites were selected to place the radon monitor, such as basements, away from doors and windows, to record the maximum radon concentration. The detectors were always placed at a height of approximately 1 m above the ground (Anastasiou et al., 2003; Theodoulou et al., 2012).

Two 24 h measurements were obtained in each defined grid and the average value was recorded as the radon concentration value for the grid. Two measurements in each grid were conducted in different seasons of the year, so no seasonal corrections were applied.

Passive alpha track detectors were exposed for mostly 2–3 months, but also for the one year period in Croatia (Radolić et al., 2006), Denmark (Andersen et al., 2007, 2001), Finland (Valmari et al., 2010; Weltner et al., 2002), Greece (Nikolopoulos et al., 2002), Hungary (Hámori et al., 2006) Iceland (Jónsson et al., 2015), Ireland (Fennell et al., 2002), Italy (Bochicchio et al., 2005; Carelli et al., 2009) and Netherlands (Lembrechts et al., 2001; Stoop et al., 1998). Electrets were used in Austria (Friedmann, 2005) with time of exposure of 3 months; in Lithuania with minimum 3 weeks (Morkunas and Akelbrom, 1999); in Hungary with one year period of exposition (Nikl, 1996) and Switzerland for 3 months (Kropat et al., 2014).

Due to the method specificity, measurements with charcoal canisters lasted for few days, the most often three to four days.

Solid state track detectors, as well as charcoal canisters were mostly placed in pairs, at least 1 m above the ground, away from door and windows, in most cases in basement and in one room on the ground floor, or one in a bedroom and one in the living room or other most frequently used room. In Greece (Nikolopoulos et al., 2002) and Croatia (Radolić et al., 2006), for example, as an exception from the usual practice, one detector was used per surveyed home, but for the whole year period. The maximum number of detectors in one object, according to presented literature survey, was in Poland - 3 detectors for mean monthly concentration and 3 for mean quarterly concentrations (Przylibski et al., 2011). Thus, 12 monthly averages and 4 quarterly averages were calculated per building.

During the indoor radon survey, measurements of ambient gamma dose rate indoors were performed at the same time in Lithuania (Morkunas and Akelbrom, 1999) and Turkey (Can et al., 2012).

Uniquely, during the surveys in Finland (Valmari et al., 2010; Weltner et al., 2002) in single measurement evaluation, corrections based on the outdoor temperature and wind speed were taken into account.

Correction factor values were mainly took from the literature, but in some countries, like Albania and Austria (Bode Tushe et al., 2016; Friedmann, 2005) the correction factors were obtained by studying the variations in indoor radon concentration observed in summer and winter seasons with respect to the entire year in randomly selected dwellings located in different geographical regions. Different approach was chosen in Czech Republic where the seasonal corrections were calculated on the basis of the data of Moucka including 3000 weekly measurements in 24 objects in the Czech Republic (Slezáková et al., 2013).

Whole year measurements were performed in at least 12 European countries. In most cases, a single detector was exposed for approximately 1 year. In other cases, 2 detectors were deployed in consecutive 6 months periods (Italy (Bochicchio et al., 2005), Malta (Baluci et al., 2013) and Montenegro (Vukotic et al., 2018)) or 4 detectors in consecutive 3 months periods (Macedonia (Stojanovska et al., 2012)). In at least 10 surveys, measurements were performed only during winter or during the heating season. This period of year was often selected in Scandinavian and Baltic countries. Other surveys were performed at least partly outside the heating season, or the time of year was not specified in the literature source. Radon concentration variability in periods longer than 1 year was widely neglected, with notable exceptions (Slezáková et al., 2013).

## 4 Sampling procedure, sampling number and type of locations

Due to its long half-life, radon is assumed to be uniformly distributed within the room. Therefore, a detector can be placed at any position in a room, exposed to air. Nevertheless, due to the change of physical properties of detectors when exposed to heat (Fleischer et al., 1975), it should be avoided to place detectors close to a heat source. A vicinity of windows and doors should be avoided as well. Since one of the goals of radon surveys is to obtain reliable estimation of exposure to radon, detectors should be placed in rooms with high occupancy such as bedrooms or living-rooms. For passive radon detectors that have substantial sensitivity to thoron it is important to place detector away from walls, in order to reduce possible contribution from thoron.

Sampling procedures in most covered surveys were similar. Mainly, two detectors were deployed per dwelling at the same time in the most frequently used rooms (like living room, kitchen or bedroom), placed away from doors and windows and one to 2 m from the floor. But there are cases, like in Greece (Nikolopoulos et al., 2002) where one detector was used, and measurement lasted for a whole year. Detectors were exposed on ground level or basements. Also, in most cases detectors were distributed with questionnaires and instructions.

A due attention should be paid to handling detectors after being exposed. They should be sealed in radon-proof bags in order to reduce unwanted overexposure of detectors, or sent immediately to responsible institution. Detailed instructions are usually sent to householders regarding the deployment and handling of the detectors after the exposure. Although improper handling of the detectors could lead to a significant overexposure, these details were not discussed in any of the reviewed articles, neither in the form of applied corrections nor in the uncertainty budget.

## 5 Data analysis

The interpretation of the bulk results was conducted, on different level, for all surveys in all countries. The results were analysed according to the survey goal and the type of the analysis depended on the survey type and strategy as well as the duration and type of measurement. In almost all papers, the basic statistical analysis, consisting of calculation of average and annual mean values, standard deviation, minimum and maximum value was performed. This basic statistics, although it cannot determine the causal links between the measured values, was able to point out the outlier results, which, on the other hand can point to the areas with untypically high values of indoor radon. In some papers, a map depicting measured or averaged results was produced. A map provides in principle the same outlook as the descriptive statistics, but in the graphic format. Also, a test for log – normality of the obtained results was performed in some studies.

Results of descriptive statistic were presented in 55 papers, describing the analysis of measurement results from 39 countries In 27 papers, covering the results of surveys in Albania, Azerbaijan, Belarus, Belgium, Bulgaria, Denmark, Finland, France, Germany, Greece, Italy, Malta, Montenegro, Netherlands, Norway, Romania, Serbia, Slovakia, Spain, Sweden, Switzerland and Ukraine, authors used the obtained average values to assess the percentile, or number of houses where the indoor radon concentration exceeded some predetermined levels (Andersen et al., 2001; Baluci et al., 2013; Bochicchio et al., 2005; Bode Tushe et al., 2016; Cinelli et al., 2011; Cucoş (Dinu) et al., 2017; Hoffmann et al., 2017; Ivanova et al., 2013; Jensen et al., 2004; Kemski et al., 2004; Nikolopoulos et al., 2002; Pavlenko et al., 2014; Poffijn et al., 1994; Sainz Fernández et al., 2017; Stoop et al., 1998; Swedjemark, 2002; Swedjemark et al., 1993; Tondeur et al., 1997; Valmari et al., 2010; Vicanova et al., 1998; Vukotic et al., 2018; Weltner et al., 2002; Yaroshevich et al., 2012; Z.S. Žunić et al., 2010a, Žunić et al., 2010b; Žunić et al., 2009). In surveys conducted in Azerbaijan, Belgium and Spain, the correlation of the results of indoor radon measurement with the geological characteristics of the region was investigated, while in Albania, the comparison with known uranium concentration in soil was performed. Also, as a form of descriptive statistics, the frequency distribution was calculated in the following surveys: Albania (Bode Tushe et al., 2016), Austria (Friedmann, 2005), Azerbaijan (Hoffmann et al., 2017) and Belarus (Yaroshevich et al., 2012).

Besides this basic analysis, in 15 papers, tests for log normality were performed. The log-normality test is performed when there is a need to analyse a set of results dependents on many independent random variables. Such is the case of indoor radon where, if the data fits the log-normal distribution, the percentage of results exceeding some threshold can be easily calculated. These tests were done for surveys in Albania (Bode Tushe et al., 2016), Belgium (Tondeur et al., 1997; Cinelli and Tondeur, 2015), Bulgaria (Ivanova et al., 2013), Croatia (Radolić et al., 2006), Hungary (Hámori et al., 2006), Ireland (Dowdall et al., 2017), Italy (Bochicchio et al., 2005), Luxemburg (Kies et al., 1997), Montenegro (Vukotic et al., 2018), Netherlands (Stoop et al., 1998), Slovenia (Križman et al., 1996), Spain (Sainz Fernández et al., 2017), Switzerland (Kropat et al., 2014) and Ukraine (Pavlenko et al., 2014).

In some surveys declustering technique were applied to reduce the effect of the over-representation in the over-sampled area (Zhu et al., 1998).

Although many of the measurement were conducted in limited time span, only in 10 papers, seasonal corrections were applied in order to make the results valid for the whole year. Depending on the survey design, measurements were conducted in the winter (heating season), thus providing the highest values of the indoor radon. In these cases, application of the seasonal indices can be omitted if conservative approach is applied. The papers where the correction with the seasonal indices was performed are covering measurements in Albania, Austria, Italy, Romania, Slovenia, Serbia and UK (Bochicchio et al., 2005; Bode Tushe et al., 2016; Cucoş (Dinu) et al., 2017; Daraktchieva et al., 2015; Friedmann, 2005; Križman et al., 1996; Miles et al., 2007, 2011; Udovičić et al., 2016). In these papers, the goal was to ascertain the indoor radon concentration throughout the whole year.

Besides statistical analysis, in some papers a map was produced. These maps were in some cases the goal of the paper and they were associated with the European indoor radon map. In other cases, the map was the means to summarise the results. In most cases, the results were depicted in the form of mean radon risk map, which integrates a variety of data available, including geological maps, radon maps, grids or measured points and administrative boundaries. Maps were produced in papers covering the survey in Austria (Friedmann, 2005), Azerbaijan (Hoffmann et al., 2017), Belgium (Cinelli et al., 2011; Poffijn et al., 1994; Tondeur et al., 1997; Zhu et al., 2001), Cyprus (Theodoulou et al., 2012), Denmark (Andersen et al., 2001), Finland (Weltner et al., 2002), Iceland (Jónsson et al., 2015), Italy (Bochicchio et al., 2005), Latvia (Dambis, 1996), Macedonia (Stojanovska et al., 2012), Malta (Baluci et al., 2013), Norway (Jensen et al., 2004), Portugal (Faisca et al., 1992), Romania (Cucoş (Dinu) et al., 2017), Russia (Zhukovsky et al., 2012), Slovenia (Humar et al., 1995; Križman et al., 1996), Spain (Sainz Fernández et al., 2017), Switzerland (Kropat et al., 2014) and UK (Daraktchieva et al., 2015; Miles et al., 2007, 2011).

## 6 Quality assurance and quality control

Quality assurance (QA) is planned and systematic action necessary to provide adequate confidence that testing or calibration will satisfy quality requirements. Quality control (QC) contains the operational techniques and activities that are used to fulfil the requirements for quality. QA and QC are necessary to avoid mistakes before they are made and to reduce uncertainties, but also help to estimate the contribution of different input quantities to the final uncertainties.

Ensuring measurement quality is usually done through metrology certification, participation in inter-comparison measurements and periodical calibrations of detectors and monitors. The results of several inter-laboratory comparison exercises showed that precision and accuracy of passive radon devices can be quite different, even for the similar or identical devices (Howarth and Miles, 2002).

Different type of QA/QC procedures for radon measurements could be carried out and the most comprehensives were reported by (Friedmann, 2005):

• Intercalibration and intercomparison exercises between different laboratories with different detector systems in a traceable radon chamber;
• Comparison of parallel measurements with different detector systems in the same homes;
• Comparison of the density distribution of the results from different detector systems used in the same area;
• Repetition of investigations in some areas during another season and by measuring other homes;
• Additional measurements in municipalities with significantly higher or lower mean radon concentration than the adjacent municipalities (cluster analysis).

Many papers describe quality assurance and quality control for radon measurements, but authors who present indoor radon survey in European countries did not pay much attention to proper description of QA and QC.

Literature overview shows that in around 30% of references, authors did not describe any quality assurance and quality control of radon and/or radon decay products measurements during the indoor radon surveys (Table 1), but some of them (France, Portugal, Spain, United Kingdom) participated in intercomparisons which were held at the National Radiological Protection Board every year. In 2003, 49 laboratories from 17 countries participated (Howarth and Miles, 2007).

**Table 1:** Reported quality assurance and quality control of radon and/or radon decay products measurements during the indoor radon surveys.

Country	Periodical calibration (or accreditation ISO 17025)	Intercalibration and intercomparison	Comparison of the results from different detector systems	Duplicate detectors	None
Albania				x	
Austria		x	X		
Azerbaijan					x
Belarus					x
Belgium		x			
Bosnia and Herzegovina					x
Bulgaria					x
Croatia					x
Cyprus	x		X		
Czech Republic	x				
Denmark					x
Estonia					x
Finland					x
France		x			
Georgia					x
Germany	x				
Greece	x				
Hungary	x				
Iceland					x
Ireland	x			x	
Italy	x	x			
Kazakhstan					x
Latvia					x
Lithuania		x			
Luxembourg					x
Macedonia	x				
Malta	x				
Moldova					x
Montenegro			X	x	
Netherlands			X		
Norway		x			
Poland		x			
Portugal				x	
Romania		x			
Russia	x		X		
Serbia					x
Slovakia	x				
Slovenia	x	x			
Spain		x			
Sweden	x				
Switzerland					x
Turkey	x	x			
Ukraine	x	x			
United Kingdom		x			

Periodical calibration of detectors or calibration through accredited laboratory services (accreditation according to ISO 17025) are the most common methods of quality control of measurement (Table 1).

Many countries have a system for calibration. In Belgium the calibration of the detectors was controlled by using two small radon reference chambers at ISIB and at the Ghent University (Tondeur, 1998). The detectors were calibrated in radon chamber at the Federal Office for Radiation Protection for measurements in Germany (Kemski et al., 2004), at the University of Athens for measurements in Greece (Nikolopoulos et al., 2002), in the reference radon and radon progeny measuring chamber at the State Metrological Centre of IPCM for the measurement in Slovakia (Vicanova et al., 1998). In Sweden the role of the SSI is to co-ordinate the work on radon and to be responsible for the calibration of measuring devices (Swedjemark, 2002).

In Ireland two radon detectors were placed per home. On return to the laboratory, the detectors were analysed using the Ireland's Environmental Protection Agency's Radon and Radiation Measurement Services test procedures which are accredited to ISO 17025 by the Irish National Accreditation Board (Dowdall et al., 2017).

In Ukraine laboratory used the quality assurance system for the indoor radon measurements which has been developed and implemented at the State institution The Marzeev Institute of Hygiene and Medical Ecology (Pavlenko et al., 2014). The quality assurance procedures included calibration of radon track detectors using the secondary calibration source of laboratory which is accredited by the National Standardization and Accreditation Authority of Ukraine.

Some countries use calibration facilities from other countries. For measurements in Hungary calibration was performed in Swedish Radiation Protection Institute (Nikl, 1996) and at NPRB in United Kingdom (Hámori et al., 2006). In Cyprus calibration over the whole dynamic range of the instrument is made and the accuracy of the calibration is then verified by the State Metrological Institute of the Czech Republic (Anastasiou et al., 2003; Theodoulou et al., 2012). In Italy, measuring system calibration was obtained by exposing a total of nine groups of radon passive devices in the radon chambers of the Heath Protection Agency, UK, and the Italian National Metrology Ionizing Radiation Institute (Carelli et al., 2009).

In Macedonia detectors exposed to known radon concentrations were used for the purpose of quality control of the system. They used full equipment, together with the detectors, the exposed detectors and the proper calibration factors which were commercially available from Hungary (Stojanovska et al., 2012).

After exposure, some countries sent detectors back to the manufacturer for reading, in a vacuum sealed plastic packages to prevent radon contamination during the travel (Serbia: (Udovičić et al., 2016); IAEA SRB/9/006, 2018). In Malta, retrieved detectors were analysed by a Health Protection Agency-accredited laboratory in UK (Baluci et al., 2013). In Russia the two versions of radon radiometers were calibrated in a radon calibration facility of the State Metrological Institute (Marenny et al., 1996).

Intercalibration and intercomparison exercises between different laboratories with different detector systems were also used. In Czech Republic the calibration was done through authorized metrological centre and verified internationally (Thomas et al., 2004) while in Belgium a long-term measurement were gathered by several Belgian laboratories, as well as through the participation in European intercomparisons (Howarth and Miles, 2007).

The measuring system has been tested through intercomparisons on national or international level in Italy (Bochicchio et al., 2005), Lithuania (Morkunas and Akelbrom, 1999), Norway (Jensen et al., 2004), Romania (Cucoş (Dinu) et al., 2017), Slovenia (Vaupotič, 2003), Spain (Sainz Fernández et al., 2017), Turkey (Köksal et al., 2004) and Ukraine (Pavlenko et al., 1997).

In Slovenia, all measuring devices have been regularly checked at the intercomparison experiments in order to comply with the QA/QC requirements, organized annually by the Slovenian Nuclear Safety Administration or by participation in the international intercomparison experiments in Austria and in Czech Republic (Humar et al., 1995; Vaupotič, 2003).

In order to make it possible to compare and compile the results obtained in several laboratories in Poland, a comparative experiment was carried out at CLOR (Mamont-Cieśla et al., 2010; Przylibski et al., 2011).

Duplicate measurements were also used for QC. Whenever possible, measurements were performed twice in each house in Portugal (Faisca et al., 1992). In Albania (Bode Tushe et al., 2016) for quality control purposes, duplicate detectors were placed in randomly selected dwellings while in Montenegro two dosimeters were placed together at each 10th measuring location (Vukotic et al., 2018).

In some surveys, beside the main passive radon detector a passive or active radon monitoring devices from other institute were used as an intercomparison result, for example in Montenegro, devices from Austria were used (Vukotic et al., 2018).

In Netherlands national surveys two type detectors were used. For the purpose of comparison the new survey with the previous one, the instruments and procedures applied in both surveys were compared (Stoop et al., 1998).

In Portugal the repetition of investigations in some areas was done during a different season (Faisca et al., 1992).

## 7 Thoron measurements

The results of radon measurements without radon-thoron discrimination might be overestimated if the detector is sensitive to thoron and the measurement is made by devices with no radon-thoron discrimination capability, such is a CR-39 detector (Nikezić and Yu, 1998). Therefore, the alpha-activity of thoron was measured at the same time as radon by closed CR-39 track detectors in Hungary (Hámori et al., 2006).

The short half-life of thoron limits the thoron exhalation from soil and building materials and thus the contribution of thoron to the radiation exposure of the population. For a good estimation of the radon and thoron doses, measurements of radon, thoron and their progeny concentrations should be carried out simultaneously (Janik et al., 2013).

The focus in indoor radon surveys is on ^222^Rn, which gives the highest doses, so in over the 70% of surveyed papers thoron was not mentioned, while some authors have written that they did not correct measurements for possible errors due to thoron concentrations (Kropat et al., 2014).

In Italy, a national survey was conducted with detectors enclosed in a heat-sealed low density polyethylene bag, which blocks radon decay products and thoron (Bochicchio et al., 2005).

In Russia the exposure to thoron progeny is not considered to be an important problem in comparison with the radon progeny (Yarmoshenko et al., 2015).

Although in many indoor radon surveys thoron is not mentioned, there are lot of papers on local radon surveys, which describe that the indoor thoron levels are significant and should be taken into account during both radon measurements and radiation dose and risk assessment, for example in some regions of Balkan: south-eastern Serbia, Kosovo and Metohija and parts of Western Serbia (Žunić et al., 2009).

The RADUET detector was used for simultaneous measurement of the radon and thoron activity in the Visegrad countries (Hungary, Poland and Slovakia), Macedonia, Serbia and Bosnia and Herzegovina (Ćurguz et al., 2015; Mú́;llerová et al., 2014; Stojanovska et al., 2012; Z.S. Žunić et al., 2010a, Žunić et al., 2010b). Detector consisted of two detector CR-39, fixed in the pot section of two diffusion chambers. The main diffusion chamber was sensitive to radon and the secondary chamber was sensitive to both radon and thoron.

In Austria the thoron progeny measurements were made in some houses in an area with a relatively high thorium concentration. Because in all cases except one, the mean effective dose of thoron progeny was less than 20% of that from radon progeny, the author concludes that the contribution of thoron to the effective dose can be neglected in most cases in Austria (Friedmann, 2005).

Some authors have estimated that thoron activity concentration is very low, but it was used for dose estimation (Yaroshevich et al., 2012).

## 8 Conclusion

The literature survey has shown that indoor radon surveys were performed in most European countries and in many cases the surveys covered the whole countries. Methodologies used in the surveys were very diverse, to such extent that it is impossible to find two complete same methodologies. This diversity makes comparison between different surveys difficult and likewise makes difficult compiling the data to produce an overall European radon map. Many sources omit some critical information on survey design, which makes it hard to evaluate the methodology or to replicate it. It was found that only in a few papers from the literature survey; authors have paid attention to the representativeness of the performed survey.

It would be very beneficial to create a uniform or at least recommended methodology for surveys aimed at contributing to European radon map and for surveys sponsored by national or international (such as International Atomic Energy Agency) authorities.

The reliability of radon measurement requires that laboratories producing analytical data are able to provide results of the required quality. The need for uniform results from laboratories at an international level therefore requires the implementation of a quality assurance programme, the harmonisation of criteria, sampling procedures, calculations and the reporting of results, agreed on the basis of fundamental principles and international standards. Due to 2013 BSS Directive more radon surveys and related work will be performed in the future and thus harmonisation and standardised methodology would be helpful.
